# Supplementary material for: Single-cell transcriptome analysis defines heterogeneity of the murine pancreatic ductal tree
Source: eLife. 2021 May 19;10:e67776. doi: 10.7554/eLife.67776 (PMC8184217; doi:10.7554/eLife.67776)
Supplement: Supplementary file 1. — This table depicts a summary of expression scoring of selected markers for subpopulations of pancreatic duct cells in mouse and human tissue. Homogeneous refers to an observed uniform expression level and pattern within a particular ductal cell type. Heterogeneous means that either the observed expression level or pattern varies among cells within a particular ductal cell type. [file elife-67776-supp1.pptx]

## Slide 1
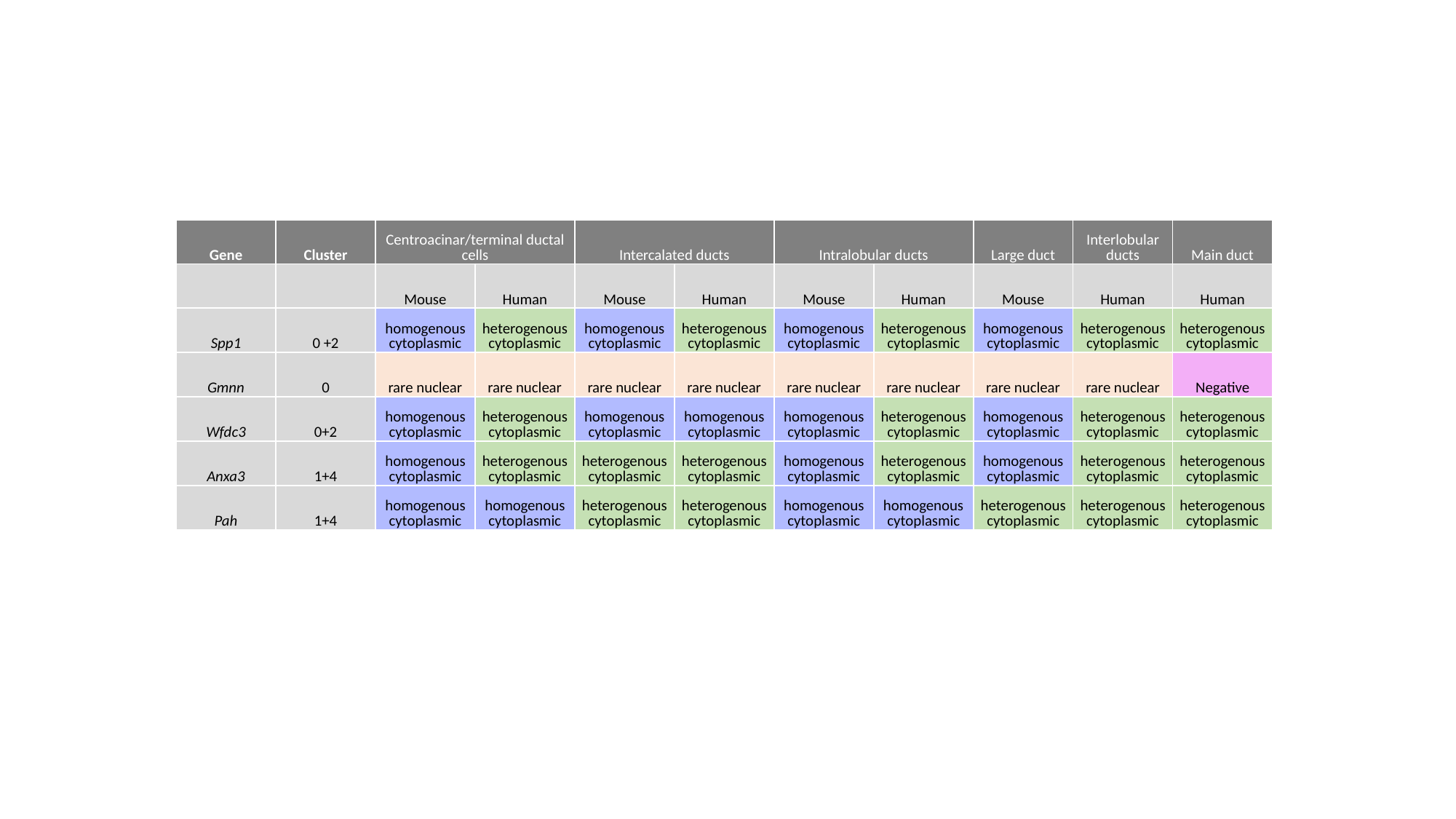

| Gene | Cluster | Centroacinar/terminal ductal cells | | Intercalated ducts | | Intralobular ducts | | Large duct | Interlobular ducts | Main duct |
| --- | --- | --- | --- | --- | --- | --- | --- | --- | --- | --- |
| | | Mouse | Human | Mouse | Human | Mouse | Human | Mouse | Human | Human |
| Spp1 | 0 +2 | homogenous cytoplasmic | heterogenous cytoplasmic | homogenous cytoplasmic | heterogenous cytoplasmic | homogenous cytoplasmic | heterogenous cytoplasmic | homogenous cytoplasmic | heterogenous cytoplasmic | heterogenous cytoplasmic |
| Gmnn | 0 | rare nuclear | rare nuclear | rare nuclear | rare nuclear | rare nuclear | rare nuclear | rare nuclear | rare nuclear | Negative |
| Wfdc3 | 0+2 | homogenous cytoplasmic | heterogenous cytoplasmic | homogenous cytoplasmic | homogenous cytoplasmic | homogenous cytoplasmic | heterogenous cytoplasmic | homogenous cytoplasmic | heterogenous cytoplasmic | heterogenous cytoplasmic |
| Anxa3 | 1+4 | homogenous cytoplasmic | heterogenous cytoplasmic | heterogenous cytoplasmic | heterogenous cytoplasmic | homogenous cytoplasmic | heterogenous cytoplasmic | homogenous cytoplasmic | heterogenous cytoplasmic | heterogenous cytoplasmic |
| Pah | 1+4 | homogenous cytoplasmic | homogenous cytoplasmic | heterogenous cytoplasmic | heterogenous cytoplasmic | homogenous cytoplasmic | homogenous cytoplasmic | heterogenous cytoplasmic | heterogenous cytoplasmic | heterogenous cytoplasmic |
